# Self-efficacy for coping: utility of the Cancer behavior inventory (Italian) for use in palliative care

**DOI:** 10.1186/s12904-019-0420-y

**Published:** 2019-04-05

**Authors:** Samantha Serpentini, Paola Del Bianco, Andrea Chirico, Thomas V. Merluzzi, Rosalba Martino, Fabio Lucidi, Gian Luca De Salvo, Leonardo Trentin, Eleonora Capovilla

**Affiliations:** 1 Istituto Oncologico Veneto IOV – IRCCS, Via Gattamelata, 64, 35128 Padova, Italy; 2 “La Sapienza” University, Rome, Italy; 3 University of Notre Dame, South Bend, Indiana, USA

**Keywords:** Self-efficacy, Coping, Oncology, Advanced cancer, Palliative care, Cancer behavior inventory

## Abstract

**Background:**

Newer models of palliative and supportive cancer care view the person as an active agent in managing physical and psychosocial challenges. Therefore, personal efficacy is an integral part of this model. Due to the lack of instruments in Italian to assess coping self-efficacy, the present study included the translation and validation of the Italian version of the Cancer Behavior Inventory–Brief (CBI-B/I) and an initial analysis of the utility of self-efficacy for coping in an Italian sample of palliative care patients.

**Methods:**

216 advanced cancer patients who attended palliative care clinics were enrolled. The CBI-B/I was administered along with the European Organization for Research and Treatment of Cancer Quality of Life Questionnaire-Core 30 (EORTC QLQ-C30), the Mini Mental Adjustment to Cancer Scale (Mini-MAC), the Cancer Concerns Checklist (CCL), and the Hospital Anxiety and Depression Scale (HADS). The Eastern Cooperative Oncology Group Performance Status (ECOG-PS) ratings of functional capacity were completed by physicians.

**Results:**

Factor analysis confirmed that the structure of the CBI-B/I was consistent with the English version. Internal consistency reliability and significant correlations with the EORTC QLQ-C30, Mini-MAC, and HADS supported the concurrent validity of the CBI-B/I. Differences in CBI-B/I scores for high versus low levels of the CCL and ECOG-PS supported the clinical utility of the CBI-B/I.

**Conclusions:**

The CBI-B/I has strong psychometric properties and represents an important addition to newer model of palliative and supportive care. In order to improve clinical practice, the CBI-B/I could be useful in identifying specific self-efficacy goals for coping in structured psychosocial interventions.

## Background

Self-efficacy theory has a long history of guiding assessment and interventions in many health domains [1–4], including cancer [5]. Generally, greater self-efficacy for coping has been positively associated with adjustment to cancer, quality of life (QOL), positive mood, and treatment seeking [5–7] and negatively correlated with cancer symptoms [8, 9]. There is an increasing interest in the role of self-efficacy with regard to patients with advanced cancer and those in palliative and supportive care, that is, those who have incurable cancer and may not be receiving medical treatment to extend life.

The evidence for the importance of self-efficacy for coping in the context of palliative and supportive care is connected to its relationship with not only self-care but also with adjustment and quality of life. For example, Baile, Palmer, Bruera, & Parker [10] found negative correlations between number of concerns (e.g., needing more information about illness/treatment, not being able to do usual activities, caring for self) and coping self-efficacy in palliative care patients. In patients with advanced disease, cancer self-efficacy expectations, which included coping self-efficacy, activities of daily living (ADL) efficacy, and affect regulation self-efficacy, mediated the relationship between functional status and emotional well-being, thus mitigating the negative effects of physical limitations on emotional well-being [11]. Similarly, a mediating effect of coping self-efficacy in the relationship between symptoms and depression was also found in a sample of cancer survivors who were on the average 9.3 years post diagnosis [12].

In order to assess the utility of coping self-efficacy in palliative and supportive care, it is critical to have established measures with good psychometric properties. One option is to develop measures that are specifically related to coping in particular phases of cancer (e.g., diagnosis, transition to survivorship, etc.). Another approach is to test the utility of measures used in all phases of care. Thus, measures of self-efficacy expectations for coping, which have been useful in assessing patients and survivors [5, 13] may also be useful for assessing those in palliative and supportive care in terms of the relationship between coping and quality of life.

In line with self-efficacy theory and supporting the need for self-efficacy-based assessments are emerging models of care for persons receiving palliative and supportive care [14]. In those models, people are not viewed as passive recipients of care but as active agents in terms of negotiating the challenges that they confront. Therefore, the concept of personal efficacy [15], particularly self-efficacy for coping, is an integral part of this new model of palliative care and, in addition, that sense of agency can be cast in the context of a general model of self-regulation [16]. That is, the process of self-regulation including having goals, making plans to reach those goals, and coping with the challenges that arise in the process are all-important aspects of quality of life in palliative and supportive care.

The challenges that advanced cancer patients may face in palliative care include dealing with severe symptoms of pain and fatigue, and having the functional capacity to manage their lives independently. Also, patients receiving palliative and supportive care may have more basic, proximal and short-term QOL goals involving specific physical and emotional challenges [17]. For example, depression represents the most common mental health problem in advanced cancer and palliative care [18] adding a significant burden to this population [19]. In addition, there are challenges in patient care with regard the difficulty patients and physicians face in engaging in discourse about dying and death [20] and providing care that respects the dignity of the person receiving it [21]. In the context of these physical and emotional challenges to the quality of life and the complexities inherent in relationships with health care providers, emerging model of palliative care in which patients are viewed as active agents in their self-care, self-efficacy expectations for particular coping behaviors are critical in striving toward QOL goals. Thus, it becomes important not only to conceptualize this emerging model of palliative and supportive care but also to be able to accurately assess and empirically describe patients in terms of this new model. Finally, this assessment process should have clinical utility for developing interventions for those who are struggling with poor quality of life and who may not easily adopt the personal agency model in palliative care.

The Cancer Behavior Inventory–Brief (CBI-B) version [5], derived from the original, longer 33-item version [13], represents a comprehensive and efficient brief measure of self-efficacy for coping with cancer that could be easily used as a patient-reported outcome measure in a palliative care clinical setting. Unlike other measures of self-efficacy for coping such as by Lev et al. [22], the CBI-B has a stable factor structure across various types of cancer as well as established psychometric qualities. Compared to a measure by Telch & Telch [23], which lacks information on its construction and psychometric analyses and has not been subjected to peer review nor published, the development of the CBI-B was clearly presented and subjected to the rigors of the publication process. The CBI-B is also much briefer than those measures and, therefore more useful in clinical research settings. Therefore, the first aim of this study was to use standard methodology to translate the CBI-B into Italian and then to confirm the structure and psychometric quality of an Italian version of the Cancer Behavior Inventory–Brief (CBI-B/I) in palliative care. The second aim was to advance the research [6, 24] on clinical utility of CBI-B/I in an Italian sample of palliative care patients by investigating scores on the CBI-B/I in relation to other measures that are important in the context of palliative and supportive care and represent targets of interventions to improve patients’ quality of life.

## Methods

### Translation of the Cancer behavior inventory-brief/Italian

The CBI-B/I is a measure of self-efficacy for coping in cancer patients. The instrument consists of 12 items (rated 1 = not at all confident to 7 = totally confident) and was derived from the longer version of the CBI [13]. As in the CBI-B English version [5], the individual items are summed to yield 4 scales (“Coping and stress management” - 3 items, “Maintaining independence” - 4 items, “Managing affect” - 3 items, and “Participating in medical care” - 2 items) and a total score or composite index. The translation of the CBI-B followed forward and backward translations of the original scale, following the EORTC translation guidelines [25]. Two Italian translators independently completed the forward translation and negotiated any differences in the two versions. The reconciled Italian version was then given to two English translators, who independently back-translated the measure. Any discrepancies were discussed and resolved, and modifications were made in the CBI-B/I to take into account any rewording to improve the conceptual relevance and comprehension of the items. Finally, a small focus group of 5 patients was convened, the resulting Italian CBI-B/I was administered, and based on the discussion of each item, final and minor modifications were made.

### Validation procedure

This study took place in three Italian cancer centers: Padua, Bassano del Grappa (Vicenza), and Naples. The sample consisted of a consecutive series of patients attending the Oncology Outpatient clinics for symptom control and palliative care. Criteria for inclusion were: age ≥ 18 years, a diagnosis of advanced cancer in which any treatment that was being administered had no curative intent, and the ability to speak and read Italian fluently. Patients were excluded from treating clinicians if they displayed any signs of cognitive impairment or had such poor functional capacity that they could not participate in the interview portion of the study.

Upon arrival to outpatient oncology clinics, patients were asked if they would be interested in participating in a research project. If they agreed, the study was explained to them, and informed consent as well as permission to obtain specific information from the participants’ medical record, were obtained. Following informed consent, the participants were interviewed individually in a private room by the researcher obtaining socio-demographic information such as age, gender, marital status, education, occupation, religious practices. Then participants were asked to complete a survey that contained the following instruments: the CBI-B/I, the EORTC QLQ-C30, the Mini-MAC, the HADS Scale, the CCL, and a short debriefing questionnaire to define patient acceptability and understanding.

### Measures

#### Concerns checklist (CCL)

The CCL [10] is a 15-item survey that is used in palliative and supportive care in which patients rate the degree of concern on a “0” (not at all) to “3” (very much) scale. Eleven items are listed (e.g., worries or concerns about the future, caring for yourself, the way doctors and nurses communicate with you) and 4 items have space for the participant to name additional concerns and rate them. The score for this study consisted of the sum of the ratings of the first 11 concerns.

#### Mini mental adjustment to Cancer scale (Mini-MAC)

The Mini-MAC [26, 27] is a 29-item instrument that evaluates cognitive and behavioral responses to cancer. The factors of the Mini-MAC are: Fighting Spirit, Hopelessness, Anxious Preoccupation, Fatalism, and Cognitive Avoidance. Each item is rated on a 4-point scale that ranges from “definitely does not apply to me” to “definitely applies to me”. Cronbach’s alphas for each scale have ranged from 0.62–0.88 [28].

#### Hospital anxiety and depression scale (HADS)

The HADS [29] is a 14-item self-report measure designed to assess depression and anxiety. Respondents are asked to rate each statement in considering the previous week on a 0–4 scale that taps into frequency. Internal consistency values for the current study were 0.85 for the depression scale, 0.83 for the anxiety scale, and 0.89 for the total scale.

#### European Organization for Research and Treatment of Cancer quality of life questionnaire-Core 30 (EORTC QLQ-C30)

The EORTC QLQ-C30 [30] includes 30 items that are rated on a 4-point scale that ranges from “not at all” to “very much”. The EORTC QLQ-C30 has a global score, 5 functional scales (physical, role, emotional, cognitive and social), 3 symptoms scales (fatigue, nausea/vomiting, pain), and 6 single items (dyspnea, insomnia, appetite loss, constipation, diarrhea and financial difficulties). For the global score and the functional scales of an Italian version of the EORTC QLQ-C30 [31], internal consistency ranged from 0.64–0.90; alphas were 0.85 for fatigue, 0.82 for pain and 0.54 for nausea/vomiting.

#### Eastern cooperative oncology group performance status (ECOG-PS)

ECOG-PS ratings were obtained from the medical records with the participants’ written permission. The ECOG-PS ratings range from “0” Fully active, able to carry on all pre-disease performance without restriction to “5” Dead. There is evidence that reliability of description is quite high when dividing patients into two groups: low (0–2) versus high [3, 4] [32].

#### Socio-demographic and medical data

Socio-demographic data were obtained from patients via an interview. Medical data obtained from the participants’ medical records included the following: ECOG-PS, diagnosis, and treatments.

### Statistical analyses

The sample size was based on the ability to verify an adequate fit of CBI-B/I to the hypothesized four-factor model of the English version [5], with 12 manifest variables. Using the root-mean-square error of approximation (RMSEA) as the measure of model fit, a minimum of 210 patients provides a 90% power level to test RMSEA≤0.05 when RMSEA = 0.08, using a 0.05 significance level [33].

Clinical and demographic characteristics of patients were described using median and range for quantitative data and frequencies and percentages for categorical data. A confirmatory factor analysis with maximum likelihood estimation was carried out to confirm the factor structure of the CBI-B/I and determine the model fit. According to Browne and Cudeck [34], a RMSEA value of < 0.05 is indicative of close fit, between 0.05 and 0.08 fair fit and > 0.10, mediocre fit. We used the non-normed fit index (NNFI) and the comparative fit index (CFI) with values > 0.9 as indicative of an acceptable model. Internal reliability was confirmed by a Cronbach alpha value greater than 0.7.

Different levels of measurement invariance were tested using a structural equation modeling approach; metric, scalar and strict invariance were verified by comparing each model with the configural invariance one. A non-significant difference in chi-squared values and a difference in CFI and RMSEA < 0.01 were considered as evidence of measurement invariance.

Concurrent validity was determined by examining the hypothesis that the higher the self-efficacy the higher the quality of life of the patients: the EORTC QLQ-C30 global score, emotional, role, social and physical functioning should positively correlate with the four factors and the composite index of CBI-B/I. The CBI-B/I should negatively correlate with anxious preoccupation (Mini-MAC) and the HADS questionnaire scales to verify that higher self-efficacy was indicative of a lower anxiety and depression. To measure concurrent validity Pearson coefficients were computed.

To test the clinical utility of the CBI-B/I, non-parametric analyses of variance were used for each dimension to verify whether the scales could differentiate between patients according to their clinical characteristics. Variables of primary interest were age, gender, spirituality, the CCL and ECOG-PS. Age was dichotomized at the median, ECOG-PS was dichotomized as 0–2 (high functioning) or 3–4 (low functioning) based on prior research [32]. Results were reported with their 95% confidence intervals (CI). All *P* values were two-sided, and P value of < 0.05 was considered statistically significant. Statistical analyses were performed using SAS statistical package (SAS, rel. 9.4; SAS Institute Inc.)

## Results

From June 2012 to July 2014, 216 advanced cancer patients were enrolled from 3 centers in Italy. Among them (median age: 60.2 years; range: 35–86), the most prevalent diagnosis was breast cancer (44.4%); the time since diagnosis was < 1 year for 28.7%, 1–5 years for 43.5%, > 5 years for 27.8% of patients; 143 (66.2%) were female, 77% married, and, for most patients (199, 92.1%), the ECOG-PS grade was 0–2 (Table 1).

**Table 1:** Clinical and demographic characteristics of patients

		Frequency	Percentage
Age	Median (range)	62 (35–86)	
Gender	Female	143	66.2
	Male	73	33.8
Marital status	Unmarried	46	22.0
	Married	163	78.0
	Missing	7	
Education	Primary/Secondary school	122	60.4
	High school/University	80	39.6
	Missing	14	
ECOG Performance Status	0–2	199	92.6
	3–4	16	7.4
Cancer site	Breast	96	44.4
	Lung	27	12.5
	Digestive/Gastrointestinal	51	23.6
	Other	42	19.4
Time since diagnosis	< 1 year	60	28.7
	1–5 years	91	43.5
	> 5 years	58	27.7
	Missing	7	

Patients reported that the CBI-B/I was brief and clear, most of them (89%) took less than 10 min to complete the questionnaire and less than 4% found some question confusing, upsetting or irrelevant.

One hundred and ninety six patients answered all items (90.7%) and only three patients missed more than two items (1.4%). Missing data were more frequent for item 12 that is inherent to the management of nausea and vomiting (10 patients).

### Confirmatory factor analysis and reliability

**Fig. 1:**
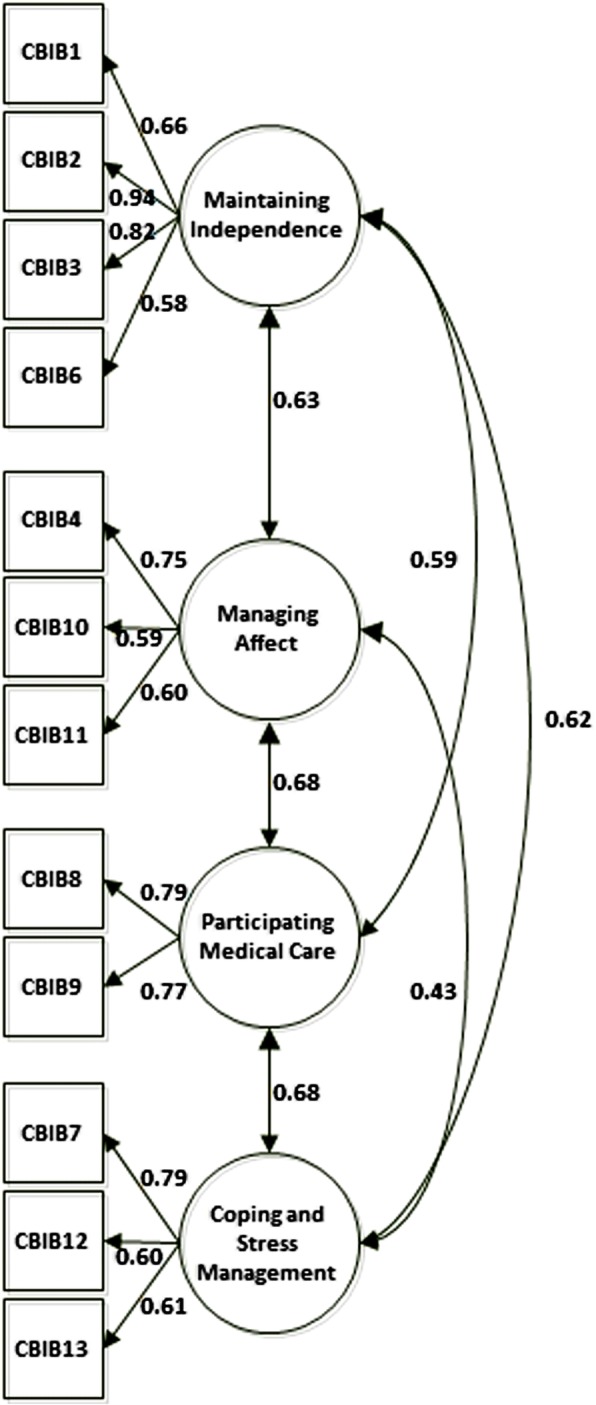
Path diagram of the four-factor structural equation model for the Italian version of the Cancer Behavior Inventory-Brief (CBI-B/I)

The hypothesized four-factor model yields a 90% confidence limit for the RMSEA with bounds of 0.06 and 0.100, providing support for a fair fit. The CFI = 0.93 and NNFI = 0.91 met all the required criteria to support the goodness of fit (Fig. 1). The paths between the items and the factors, measured by the loadings, were all statistically significant, ranging from 0.58 to 0.94, with only two slightly below 0.60, indicating that all items contributed to measurement of the underlying latent factor with a proportion of explained variance of at least 34%.

Considering the Cronbach reliability estimates for each factor (0.72 for “Coping stress management”, 0.83 for “Maintaining independence”, 0.69 for “Managing affect” and 0.75 for “Participating medical care”) only “Managing affect” was marginally below the 0.70. Importantly, the internal consistency for the composite CBI-B/I score was 0.86. Finally, correlation between factors, which were all significant, ranged from 0.43 to 0.68, indicating a moderate relationship among these constructs.

### Configural invariance

The model structure was invariant across sex, with all factor loadings statistically significant and an RMSEA of 0.09 (90% CI: 0.07–0.11), indicating that male and female patients conceptualize the construct in the same way. Comparisons to test the metric invariance, scalar invariance and strict invariance are reported in Table 2. All chi-squared tests were not significant and the difference in CFI and RMSEA between each model and the configural invariance model were < 0.01. These data indicated that the magnitude of loadings, intercepts and residual variances was similar across groups, thus confirming invariance as a function of participant sex.

**Table 2:** *Measurement invariance analysis*

		χ2	DF	*p-value*	CFI	RMSEA
Configural Invariance	Female	96.14	48		0.93	0.09
	Male	75.86	48		0.90	0.09
	Both	172.00	96		0.92	0.09
Metric invariance		187.28	108		0.91	0.09
(equal loadings)	Δ	15.28	12	*0.2263*	0.004	0.003
Scalar invariance		191.21	116		0.92	0.08
(equal loadings+intercepts)	Δ	3.93	8	*0.8634*	0.005	0.005
Strict invariance		207.00	128		0.92	0.08
(equal loadings+intercepts+residuals)	Δ	15.78	12	*0.2014*	0.004	0.002

### Demographic analyses

There were no significant correlations between the CBI-B/I and the most of socio-demographic data (i.e., sex, time since diagnosis, marital status, spirituality). Regarding age, we found that older patients (> 62 years old) reported higher self-efficacy than patients with less than 62 (*p* = 0.0225), which was consistent with other versions of the CBI [13]. The non-significant effects for most correlation of the CBI-B/I and demographic variables is important in supporting the clinical utility of the CBI-B/I in that the only moderator variable to consider in assessing coping self-efficacy is age.

### Concurrent validity

Concurrent validity was tested by examining the significance of correlation coefficients between the CBI-B/I and measures of quality of life (EORTC QLQ-C30), responses to cancer (Mini-MAC), and distress (HADS) (Table 3). The four factors and the composite index of CBI-B/I were positively correlated with overall quality of life, as well as emotional, role, social and physical functioning. With respect to the Mini-MAC, the four factors and the composite index of CBI-B/I were positively correlated with Fighting Spirit, Fatalism, and Cognitive Avoidance and negatively correlated with Hopelessness, and Anxious Preoccupation. The four factors and the composite index of CBI-B/I were negatively correlated with the Anxiety and Depression scales of the HADS. Finally, the composite CBI-B/I was negatively correlated with the CCL (r = − 0.30). The direction and significance of these coefficients confirmed the validity of the CBI-B/I.

**Table 3:** *Concurrent validity: Correlation of the Italian version of the Cancer Behavior Inventory-Brief (CBI-B/I) with the EORTC Quality of Life Questionnaire (QLQ-C30), the Cancer Concerns Checklist (CCL), the Mini Mental Adjustment to Cancer Scale (Mini-MAC) and the Hospital Anxiety and Depression Scale (HADS)*

	*CBI-B/I*				
	Composite Index	Maintaining Independence	Managing Affect	Participating Medical Care	Coping Stress Management
*EORTC QLQ-C30*					
Global Index	0.26	0.28	0.19	0.17	0.20
*p-value*	*<.0001*	*<.0001*	*0.0047*	*0.0106*	*0.0037*
Emotional Functioning	0.36	0.36	0.21	0.26	0.35
*p-value*	*<.0001*	*<.0001*	*0.0027*	*0.0001*	*<.0001*
Role Functioning	0.27	0.31	0.15	0.21	0.205
*p-value*	*0.0001*	*<.0001*	*0.0238*	*0.0022*	*0.003*
Social Functioning	0.29	0.20	0.24	0.26	0.305
*p-value*	*<.0001*	*0.0031*	*0.0004*	*0.0001*	*<.0001*
Physical Functioning	0.24	0.29	0.10	0.15	0.235
*p-value*	*0.0005*	*<.0001*	*0.13*	*0.0251*	*0.0006*
Fatigue	−0.23	−0.26	−0.08	−0.20	−0.24
*p-value*	*0.0008*	*0.0001*	*0.2766*	*0.0032*	*0.0005*
Pain	−0.14	− 0.17	− 0.05	− 0.12	− 0.10
*p-value*	*0.0410*	*0.0127*	*0.4830*	*0.0840*	*0.1287*
Dyspnea	−0.19	− 0.12	− 0.10	−0.22	− 0.22
*p-value*	*0.0055*	*0.0816*	*0.1459*	*0.0011*	*0.0013*
Appetite loss	−0.21	− 0.24	− 0.02	−0.22	− 0.21
*p-value*	*0.0025*	*0.0003*	*0.7016*	*0.0014*	*0.0025*
*CCL*	−0.30	−0.24	− 0.18	−0.25	− 0.36
*p-value*	*<.0001*	*0.0005*	*0.0069*	*0.0002*	*<.0001*
*Mini-MAC*					
Fighting Spirit	0.42	0.38	0.28	0.30	0.315
*p-value*	*<.0001*	*<.0001*	*<.0001*	*<.0001*	*<.0001*
Hopelessness	−0.46	−0.47	−0.31	−0.30	−0.32
*p-value*	*<.0001*	*<.0001*	*<.0001*	*<.0001*	*<.0001*
Fatalism	0.23	0.08	0.19	0.22	0.27
*p-value*	*0.0006*	*0.2519*	*0.0055*	*0.0013*	*<.0001*
Anxious Preoccupation	−0.29	−0.25	−0.24	−0.19	−0.28
*p-value*	*<.0001*	*0.0002*	*0.0004*	*0.0058*	*<.0001*
Cognitive Avoidance	0.22	0.10	0.14	0.20	0.21
*p-value*	*0.0012*	*0.1343*	*0.0445*	*0.0029*	*0.0018*
*HADS*					
Anxiety	−0.40	−0.38	−0.23	−0.26	−0.42
*p-value*	*<.0001*	*<.0001*	*0.0008*	*0.0002*	*<.0001*
Depression	−0.46	−0.51	−0.30	− 0.25	−0.30
*p-value*	*<.0001*	*<.0001*	*<.0001*	*0.0003*	*<.0001*

### Clinical utility of the CBI-B/I for supportive and palliative care

The clinical utility of the CBI-B/I was investigated by comparing the composite CBI-B/I score at high and low levels on the CCL and the ECOG-PS. Because this utility analysis is based on different methods of assessment (CCL: self-report, ECOG-PS: physicians’ ratings), the convergence of findings from these two measures would augment concurrent validity as well as provide evidence for the clinical utility of the CBI-B/I. The mean differences on the composite CBI-B/I index were computed for those patients who, on the CCL, reported 3 or less concerns compared those who reported more than 3. Those with less concerns reported significantly higher efficacy scores. Similar to the CCL, those with higher functional status (ECOG-PS: 0–2) reported significantly higher coping efficacy (Table 4). The convergence of the results for both the CCL and the ECOG-PS contributes to the potential clinical utility of the CBI-B/I for use in palliative and supportive care.

**Table 4:** *Clinical Utility Analyses of the Italian version of the Cancer Behavior Inventory-Brief (CBI-B/I) using the Cancer Concerns Checklist (CCL) and the ECOG Performance Status (ECOG-PS)*

		*CBI-B/I Composite Index*						
		N	Mean	SD	Median	Lower Quartile	Upper Quartile	*p-value*
CCL	≤3 concerns	32	83.66	15.97	84.00	76.50	95.50	*0.0013*
	> 3 concerns	180	73.46	16.66	77.50	61.50	85.50	
ECOG-PS	0–2	199	75.69	16.66	78.00	63.00	87.00	*0.0551*
	3–4	16	65.06	19.80	65.00	50.50	83.00	

## Discussion

The aim of the present study was to translate and confirm the structure, psychometric quality, and the utility in palliative care of the Italian version of the CBI-B.

The cross-cultural adaption of the CBI-B/I did not present any particular problem during the translation process of the concepts from English to Italian language. The translation procedure was linear and the translated version resulted simple, well-interpreted and easy to complete. No ambiguous terms or discrepancies between the two versions were identified and the only item with some missing answers was related to the management of nausea and vomiting, probably due to the absence of this symptoms. The translation techniques reveal an adequate conceptual equivalence and a good level of comprehensibility.

Our analyses revealed that the CBI-B/I had the same structure as the original English version [5], was reliable, valid, and has clinical utility.

The composite index of CBI-B/I was positively correlated with the overall quality of life, as well as the EORTC QLQ-C30 subscales that measure physical, emotional, role, and social functioning: these results demonstrate that palliative care patients with higher scores on self-efficacy for coping with cancer also have better quality of life. Moreover, the significant negative correlations of CBI-B/I composite index with the following EORTC QLQ-C30 symptom subscales -fatigue, pain, dyspnea, and appetite loss– highlighted an important relationship between self-efficacy for coping and symptom management. Considering the cross-sectional design of our study, we cannot infer the directional causation. Therefore, an implication of these quality of life findings could suggest that reducing symptoms could lead to improving coping but also that interventions to improve coping efficacy may affect symptom management. This should be tested in a longitudinal design.

As hypothesized, the CBI-B/I was negatively correlated with CCL, which is a proxy for emotional distress [10] and corroborates the negative relationship between the CBI-B/I and HADS, which assesses anxiety and depression. These results are similar to the English version of the CBI-B [5] and furthermore, reinforce the parallel between the English and the Italian versions.

Consistent with recent studies [28], we found that the CBI-B/I was positively correlated with Fighting Spirit, Cognitive Avoidance and Fatalism. This may indicate that, where the options for treatment to cure the disease may not be forthcoming, distraction and not thinking about one’s illness can alleviate emotional distress. In fact, distraction is widely recognized as emotion-focused coping strategy [35] for the potential to diverts the focus of the attention from negative to positive thoughts. Furthermore, in some current literature, there seem to be cultural differences emerging regarding the role of an avoidant coping response. In the Chinese, Korean as well as the Greek and Italian versions of the Mini-MAC, Cognitive Avoidance is considered to be an indicator of positive adjustment [26, 36–38], whereas in the Norwegian and English versions, Cognitive Avoidance appears to be an indicator of poor adjustment [27, 39]. Similarly, Grassi et al. [26] found that Fatalism and Cognitive Avoidance in Spanish and Portuguese cancer patients represented adaptive coping strategies.

Thus, although Fatalism was originally identified as a stoic acceptance response [40] representing maladaptive coping [41], there are some aspects of this coping method that may signify acceptance of advanced disease and could potentially be adaptive [40, 42], particularly in palliative care. Along those lines, recent analyses by the authors of the original MAC scale have shown that a more general scale of positive adjustment, in fact, included items from both the Fighting Spirit and Fatalism scales [40, 42]. Thus, the modestly positive correlation between the CBI-B/I and Fatalism may be understandable in the context of palliative and supportive care, in that the items in the Fatalism scale express a sense of “letting go” [43], and benefit finding [44], which may relieve anxiety and stress.

The second aim of the present study was to establish the utility of self-efficacy for coping by investigating the relationship of self-efficacy with constructs that are important in the context of palliative and supportive care. Whereas the data are preliminary, it does appear, with respect to patient-reported concerns (CCL) and physician-reported functional status (ECOG-PS), that there is a convergence of the relationship of clinically relevant issues and functioning, as represented by the CBI-B/I. The potential utility of the CBI-B/I as a clinical assessment measure is bolstered by these findings, but more work is needed in randomized clinical trials and longitudinal research to firmly establish the clinical utility of the CBI-B/I.

There are limitations that also might be the springboard for future work. Firstly, the cross-sectional nature of the study with only one point in time allows only comments on our results in terms of association and not causality. Also, the sample had a large number of female breast cancer patients, and because of the size, analyses could not be conducted taking into account type of cancer. Future studies could conduct analyses within diagnoses including measurement invariance as a function of type of cancer.

## Conclusions

In emerging models of palliative and supportive care, patients are encouraged to be active agents in their medical care. From this perspective, personal self-efficacy for coping represents a key concept in the management of the quality of life of patients. In order to improve clinical practice in supportive and palliative care, it would be useful to identify the specific level of self-efficacy for coping in patients receiving palliative care that could be considered clinically critical. This could be an important resource both in the identification of patients’ adaptation process and in structuring specific psychosocial interventions that are personalized and tailored. Because self-efficacy is a specific mutable factor [15] that can be facilitated with specific psychosocial treatments, it can become a focal point of interventions. Thus, high quality measures like the CBI-B/I are critical in the development and implementation of those interventions in emerging models of palliative and supportive care. This research contributes to those new directions.

## Abbreviations

ADL: Activities of Daily Living
CBI-B: Cancer Behavior Inventory-Brief
CBI-B/I: Italian version of Cancer Behavior Inventory-Brief
CCL: Cancer Concerns Checklist
CFI: Comparative Fit Index
CI: Confidence Intervals
ECOG-PS: Eastern Cooperative Oncology Group Performance Status
EORTC: European Organization for Research and Treatment of Cancer Quality of Life Questionnaire
HADS: Hospital Anxiety and Depression Scale
Mini-MAC: Mini Mental Adjustment to Cancer Scale
NNFI: Non-Normed Fit Index
QLQ-C30: Quality of Life Questionnaire-Core 30
QOL: Quality of Life
RMSEA: Root-Mean-Square Error of Approximation
